# Supplementary material for: Changing social inequalities in smoking, obesity and cause-specific mortality: Cross-national comparisons using compass typology
Source: PLoS One. 2020 Jul 10;15(7):e0232971. doi: 10.1371/journal.pone.0232971 (PMC7351173; doi:10.1371/journal.pone.0232971)
Supplement: S1 File — (PDF) [file pone.0232971.s001.pdf]

Supplementary Material

Further Methods

Mathematical derivation for the compass typology with odds ratios

The compass plots have previously been used for rates. In this paper, we also wish to use them for risk factors, with prevalence bound between 0 and 1. To avoid modelling risks less than 0 or more than 1, we also shifted to logistic regression. Accordingly, we altered the plots to show the odds of smoking on the x-axis (on a logarithmic scale) and the RII as an odds ratio from a logistic regression on the y-axis (again on a logarithmic scale). For the SII contour plots, we needed an equation to calculate the SII of odds of average smoking in the population and the OR (or the RII). The SII is the absolute difference in odds predicted from the logistic regression for SES = 1 vs SES = 0.

To estimate the SII, note that log of the odds of smoking for the hypothetical person with lowest SES is:

$$\ln[Odds_{low}] = \alpha + ln[RII]$$

where α is the intercept in the logistic regression, and RII is the odds ratio for low versus high SES. And:

$$\ln[Odds_{high}] = \alpha$$

The log odds of the average smoking prevalence in the population is that when the cumulative rank value for SES is 0.5, namely:

$$\ln[Odds_{ave}] = \alpha + 0.5 \times \ln[RII]$$

Solving for α:

$$\alpha = ln[Odds_{ave}] - 0.5 \times \ln[RII]$$

The SII will then be $(Odds_{low} - Odds_{high})$:

$$SII = exp(ln[Odds_{ave}] + 0.5 \times ln[RII]) - exp(ln[Odds_{ave}] - 0.5 \times ln[RII])$$

Rearranging to solve for odds given RII and SII:

$$odds = \frac{SII}{exp(0.5 \times ln[RII]) - \frac{1}{exp(0.5 \times ln[RII])}}$$

The above equation was used to generate the SII contour lines, keeping SII fixed and varying RII to generate the associated odds values for plotting the line.
