# Supplementary material for: Changing social inequalities in smoking, obesity and cause-specific mortality: Cross-national comparisons using compass typology
Source: PLoS One. 2020 Jul 10;15(7):e0232971. doi: 10.1371/journal.pone.0232971 (PMC7351173; doi:10.1371/journal.pone.0232971)
Supplement: S1 Table — (DOCX) [file pone.0232971.s004.docx]

**Table S1: Countries excluded because mortality data was not available**

| **Region** | **Country** | **Data** | **Years covered by the analysis** | | | | | | |
| --- | --- | --- | --- | --- | --- | --- | --- | --- | --- |
|  |  |  | **1980s** |  | **1990s** |  | **2000s** |  | **2010s** |
| Western Europe | Germany | Mortality |  |  |  |  |  |  |  |
|  |  | Smoking/obesity |  |  | 1990-92 | 1997-99 | 2002-03 | 2008-11 |  |
|  | Ireland | Mortality |  |  |  |  |  |  |  |
|  |  | Smoking/obesity |  | 1987 (smok) |  | 1998 | 2002 | 2007 |  |
|  |  | Smoking/obesity |  |  |  |  | 2003 | 2008 |  |
|  | Netherlands | Mortality |  |  |  |  |  |  |  |
| Eastern Europe | Latvia | Mortality |  |  |  |  |  |  |  |
|  |  | Smoking/obesity | 1980 (smok) | 1986 (smok) | 1990 (smok) | 1997 | 2000 | 2005,  2009 |  |
| Southern Europe | Portugal | Mortality |  |  |  |  |  |  |  |
|  |  | Smoking/obesity |  | 1987 (smok) |  | 1995-96, 1998-99 |  | 2005-06 |  |
