# Supplementary material for: Changing social inequalities in smoking, obesity and cause-specific mortality: Cross-national comparisons using compass typology
Source: PLoS One. 2020 Jul 10;15(7):e0232971. doi: 10.1371/journal.pone.0232971 (PMC7351173; doi:10.1371/journal.pone.0232971)
Supplement: S2 Table — (DOCX) [file pone.0232971.s005.docx]

Table S2: Countries excluded because mortality inequality data was not available from both the 1980s and 2000s decades.

| **Region** | **Country** | **Data** | **Years covered by the analysis** | |  |  |  |  |  |
| --- | --- | --- | --- | --- | --- | --- | --- | --- | --- |
|  |  |  | **1980s** |  | **1990s** |  | **2000s** |  | **2010s** |
| Northern Europe | Denmark~ | Mortality |  |  | 1991-95 | 1996-00 | 2001-05 |  |  |
|  |  | Smoking/obesity |  | 1987 | 1994 |  | 2000 | 2005 | 2010 |
|  | Sweden~~ | Mortality |  |  | 1990-94 | 1995-99 | 2000-04 | 2005-08 |  |
|  |  | Smoking/obesity | 1980-81 | 1988-89 |  | 1996-97 | 2004-05 |  |  |
| Western Europe | Belgium | Mortality |  |  | 1991-96 |  | 2004-05 |  |  |
|  |  | Smoking/obesity |  |  |  | 1997 | 2001, 2004 | 2008 |  |
|  | Scotland | Mortality |  |  |  |  | 2001-05 | 2006-10 |  |
|  |  | Smoking/obesity |  |  |  | 1995, 1998 | 2003 |  |  |
|  | Switzerland~~ | Mortality |  |  | 1991-95 | 1996-00 | 2001-05 | 2006-08 |  |
|  |  | Smoking/obesity |  |  | 1992 | 1997 | 2002 | 2007 |  |
|  |  | Smoking/obesity |  |  |  | 1994+2000 |  | 2006+2010 |  |
| Eastern Europe | Poland | Mortality |  |  | 1991-93 |  | 2001-03 |  |  |
|  |  | Smoking/obesity |  |  |  | 1996 | 2004 | 2009 |  |
|  | Slovenia~ | Mortality |  |  | 1991-95 |  | 2002-06 |  |  |
|  |  | Smoking/obesity | 1981-1982+ 1989* |  |  | 1994+ 1996+ 1999+ 2001 |  |  |  |
| Southern Europe | Turin (Italy)~~ | Mortality |  |  | 1991-96 | 1996-01 | 2001-06 | 2006-10 |  |
|  | Italy | Smoking/obesity | 1983 | 1987* | 1990, 1994 |  | 2000 | 2005 | 2010 |
|  | Barcelona (Spain)~ | Mortality |  |  | 1992-96 | 1997-01 | 2002-06 | 2007-10 |  |
|  | Basque Country (Spain) | Mortality |  |  |  | 1996-01 | 2002-06 |  |  |
|  | Madrid (Spain) | Mortality |  |  |  | 1996-97 | 2002-03 |  |  |
|  | Spain | Smoking/obesity |  | 1987 | 1993 |  | 2001 | 2006 | 2011 |
