# Supplementary material for: Changing social inequalities in smoking, obesity and cause-specific mortality: Cross-national comparisons using compass typology
Source: PLoS One. 2020 Jul 10;15(7):e0232971. doi: 10.1371/journal.pone.0232971 (PMC7351173; doi:10.1371/journal.pone.0232971)
Supplement: S3 Table — *162–163, 165 (ICD-9) and C33–C34, C39 (ICD-10) in most Europe studies in the 1990s, ~not included for NZ smoking related mortality, ICD coding based on that in: Mackenbach JP, Kulhanova I, Menvielle G, et al. Trends in inequalities in premature mortality: a study of 3.2 million deaths in 13 European countries. J Epidemiol Community Health. 2014;69(3):207–217. (DOCX) [file pone.0232971.s006.docx]

Table S3: ICD coding for the cause-specific mortality groups

*Mortality ICD 9 ICD 10*

Cardiovascular diseases 390–459 I00–I99

Cancers 140–239 C00–D48

Other diseases Rest (001–799) Rest (A00–R99)

External causes E800–E999 V01–Y98

Smoking-related mortality

- lung cancer 162* C33–C34*
- COPD 490-492, 494, 496 J40-J44, J47
- laryngeal cancer~ 161 C32

Notes:

*162–163, 165 (ICD-9) and C33–C34, C39 (ICD-10) in most Europe studies in the 1990s

~not included for NZ smoking related mortality

ICD coding based on that in: Mackenbach JP, Kulhanova I, Menvielle G, et al. Trends in inequalities in premature mortality: a study of 3.2 million deaths in 13 European countries. *J Epidemiol Community Health.* 2014;69(3):207-217.
