# Supplementary material for: Changing social inequalities in smoking, obesity and cause-specific mortality: Cross-national comparisons using compass typology
Source: PLoS One. 2020 Jul 10;15(7):e0232971. doi: 10.1371/journal.pone.0232971 (PMC7351173; doi:10.1371/journal.pone.0232971)
Supplement: S4 Table — North is increasing relative inequalities, south is decreasing relative inequalities, west is decreasing prevalence/mortality, and east is increasing prevalence/mortality. South West corresponds with decreasing absolute and relative inequalities and decreasing rates/prevalence. It is the most preferable trend. All countries were required to have data from the first and last decade of the prescribed time periods. The trajectory is taken to be the average linear trend over the whole time period. Where the trajectory was borderline, both possible directions were recorded in the table. Note the distance of the trajectory is not presented in this table and some changes were large and some were very small. (DOCX) [file pone.0232971.s007.docx]

Table S4: Compass trajectories for general mortality cause specific inequality trends between 1980 and 2010, and smoking and obesity inequalities between 1990 and 2010

|  | Sub-region | Country | CVD | Cancer | Injury | Smoking related mortality | Smoking | Obesity |
| --- | --- | --- | --- | --- | --- | --- | --- | --- |
| **Men** | Australasia | New Zealand | NW | NW | N | NW | NW | E |
|  | Northern Europe | Finland | NW | W/NW | NW | W | NW | E |
|  |  | Norway | NW | NW | NW | N | NW | NE |
|  | Western Europe | Austria | NW | NW | W/SW | NW | NW | E |
|  |  | France | NW | W | SW | SW | NW | E/SE |
|  |  | England (and Wales) | NW | SW | NW | NW | NW | . |
|  | Eastern Europe | Czech Republic | NW | N/NW | N | NW | N | NE |
|  |  | Estonia | N | N | NE | NW | SW | SE |
|  |  | Hungary | N | NE | NW | N | NW | SE |
|  |  | Lithuania | N | NW | NE | NW | NW | SE |
| **Women** | Australasia | New Zealand | NW | N | NW | NE | NW | NE |
|  | Northern Europe | Finland | NW | NW | N | NE | NW | SE |
|  |  | Norway | NW | NW | NW | NE | NW | SE |
|  | Western Europe | Austria | NW | W | W | NE | N/NE | E |
|  |  | France | NW | N | NW | NE | NE | NE |
|  |  | England (and Wales) | NW | SW | NW | SE | NW | . |
|  | Eastern Europe | Czech Republic | NW | N/NW | NW | N | S | E |
|  |  | Estonia | NW | NW | NE | N | N/NW | SE |
|  |  | Hungary | N/NW | N | NW | NE | N/NE | SE |
|  |  | Lithuania | N | NW | NE | N/NW | NE | NE |

Notes: North is increasing relative inequalities, south is decreasing relative inequalities, west is decreasing prevalence/mortality, and east is increasing prevalence/mortality. South West corresponds with decreasing absolute and relative inequalities and decreasing rates/prevalence. It is the most preferable trend. All countries were required to have data from the first and last decade of the prescribed time periods. The trajectory is taken to be the average linear trend over the whole time period. Where the trajectory was borderline, both possible directions were recorded in the table. Note the distance of the trajectory is not presented in this table and some changes were large and some were very small.
