# Supplementary material for: Changing social inequalities in smoking, obesity and cause-specific mortality: Cross-national comparisons using compass typology
Source: PLoS One. 2020 Jul 10;15(7):e0232971. doi: 10.1371/journal.pone.0232971 (PMC7351173; doi:10.1371/journal.pone.0232971)
Supplement: S5 Table — (DOCX) [file pone.0232971.s008.docx]

Table S5a: Characteristics of the mortality data

| **Population** | **Years** | **Type of dataset** | **Geographic**  **coverage** | **Demographic**  **coverage** | **Age-range** |
| --- | --- | --- | --- | --- | --- |
| New Zealand | 1981-84/1986-89/1991-94/1996-99/2001-06/2006-11 | longitudinal | national | whole population | 35-74y |
| Finland | 1981-85/1986-90/1991-95/1996-00/2001-05/2006-10 | longitudinal | national | 20% of Finns are excluded (at random) | 35-79y |
| Norway | 1980-85/1985-90/1990-95/1995-01/2001-06/2006-09 | longitudinal | national | whole population | 40-79y |
| Austria | 1981-82/1991-92/2001-02 | longitudinal | national | whole population | 35-79y |
| France | 1980-81/1982-86/1987-89/1990-94/1995-98/1999-03 /2004-07 | longitudinal | national | 1% of the population, born outside France mainland excluded | 35-79y |
| England & Wales | 1976-81/2001-06/2006-09 | longitudinal | national | 1% of the population | 35-79y |
| Czech Republic | 1982-85/1998-03 | cross-sectional unlinked | national | whole population | 35-79y |
| Estonia | 1987-91/1998-02 | cross-sectional unlinked | national | whole population | 35-79y |
| Hungary | 1978-81/1988-91/1999-02 | cross-sectional unlinked | national | whole population | 35-79y |
| Lithuania | 1988-90 | cross-sectional (adjusted) | national | whole population | 35-69y |
|  | 2001-05/2006-09 | longitudinal | national | whole population | 35-69y |

**Table S5b: Characteristics of the smoking data [1]**

| **Population** | **Years** | **Dataset** | **Geographic**  **coverage** | **Age-range** | **Measure** |
| --- | --- | --- | --- | --- | --- |
| New Zealand | 1996-97/2002-03/2006-07/2011-12 | New Zealand Health Survey | national^1^ | 30-79y | Daily tobacco smoking |
| Finland | 1993/1995/1997/1999/2001/ 2003/2005/2007/2009/2011 | Health Behaviour and Health among the Finnish Adult population (AVTK, up to 65yo) and among the Finnish Elderly population (EVTK, 65+yo) | national | 30-79y | Current tobacco smoking |
| Norway | 1998/2002/2005/2008 | Norwegian Level of living surveys | national | 30-79y | Current tobacco smoking |
| Austria | 1991/1999 | Micro Census | national | 30-79y | Daily tobacco smoking |
|  | 2006 | Health Interview Survey | national | 30-79y | Daily tobacco smoking |
| France | 1991 | Enquête Décennale Santé | national | 30-74y | Current tobacco smoking |
|  | 2000/2005/2010 | Baromètre santé | national | 30-74y | Current tobacco smoking |
| England | 1990/1996/2000/2005 | General Household Survey | national | 30-69y | Current tobacco smoking |
|  | 2010 | Health Survey England | national | 30-69y | Current tobacco smoking |
| Czech Republic | 1993/1999/2002 | Health Interview Survey in the Czech Republic | national | 30-79y | Current tobacco smoking |
|  | 2008 | European Health Interview Survey | national | 30-79y | Current tobacco smoking |
| Estonia | 1996/2006 | Estonian Health Interview Survey | national | 30-79y | Current tobacco smoking |
| Hungary | 1994/2000/2003 | National Health Interview Survey | national | 30-79y | Current tobacco smoking |
|  | 2009 | European Health Interview Survey | national | 30-79y | Current tobacco smoking |
| Lithuania | 1994/2000/2006/2010 | Health Behaviour among Lithuanian Adult Population | national | 30-79y | Current tobacco smoking |

Note: ^1^Only NZ results were survey-weighted

**Table S5c: Characteristics of the obesity data [2]**

| **Population** | **Years** | **Dataset** | **Geographic**  **coverage** | **Age-range** | **Measure** |
| --- | --- | --- | --- | --- | --- |
| New Zealand | 2002-03/2006-07/2011-12 | New Zealand Health Survey | national^1^ | 30-79y | Measured height and weight |
| Finland | 1993/1995/1997/1999/2001/ 2003/2005/2007/2009/2011 | Health Behaviour and Health among the Finnish Adult population (AVTK, up to 65yo) and among the Finnish Elderly population (EVTK, 65+yo) | national | 30-79y | Current tobacco smoking |
| Norway | 1998/2002/2005/2008 | Norwegian Level of living surveys | national | 30-79y | Self-reported |
| Austria | 1991/1999 | Micro Census | national | 30-79y | Self-reported |
|  | 2006 | Health Interview Survey | national | 30-79y | Self-reported |
| France | 1991 | Enquête Décennale Santé | national | 30-79y | Self-reported |
|  | 2000/2005/2010 | Baromètre santé | national | 30-79y | Self-reported |
| England | 1990/1996/2000/2005/2010 | (Not-available) |  |  |  |
| Czech Republic | 1993/1999/2002 | Sample survey of the health status of the Czech population | national | 30-79y | Self-reported |
|  | 2008 | European Health Interview Survey | national | 30-79y | Self-reported |
| Estonia | 1996/2006 | Estonian Health Interview Surveys | national | 30-79y | Self-reported |
| Hungary | 1994/2000/2003 | National Health Interview Survey | national | 30-64y | Self-reported |
|  | 2009 | European Health Interview Survey | national | 30-64y | Self-reported |
| Lithuania | 1994/2000/2006/2010 | Health Behaviour among Lithuanian Adult Population | national | 30-64y | Self-reported |

Note: ^1^Only NZ results were survey-weighted

# References

1. Hu Y, van Lenthe FJ, Platt S, Bosdriesz JR, Lahelma E, Menvielle G, et al. The Impact of Tobacco Control Policies on Smoking Among Socioeconomic Groups in Nine European Countries, 1990-2007. Nicotine Tob Res. 2017;19(12):1441-9. Epub 2016/09/11. doi: 10.1093/ntr/ntw210. PubMed PMID: 27613922.

2. Hoffmann K, De Gelder R, Hu Y, Bopp M, Vitrai J, Lahelma E, et al. Trends in educational inequalities in obesity in 15 European countries between 1990 and 2010. Int J Behav Nutr Phys Act. 2017;14(1):63. Epub 2017/05/10. doi: 10.1186/s12966-017-0517-8. PubMed PMID: 28482914; PubMed Central PMCID: PMCPMC5421333.
