# Supplementary material for: Changing social inequalities in smoking, obesity and cause-specific mortality: Cross-national comparisons using compass typology
Source: PLoS One. 2020 Jul 10;15(7):e0232971. doi: 10.1371/journal.pone.0232971 (PMC7351173; doi:10.1371/journal.pone.0232971)
Supplement: S6 Table — (DOCX) [file pone.0232971.s009.docx]

**SUPPORTING INFORMATION**

**Table S5d: Source mortality and risk factor datasets, where available**

| **Datasets** | **Country** | **Agency to which requests for data can be sent** | **Link or reference** |
| --- | --- | --- | --- |
| Mortality | New Zealand | Statistics New Zealand | https://www.stats.govt.nz/integrated-data/apply-to-use-microdata-for-research/ |
|  | Finland | Statistics Finland | <http://www.stat.fi/tup/mikroaineistot/index_en.html>. Contact by email [tutkijapalvelut@stat.fi](mailto:tutkijapalvelut@stat.fi) or by telephone +358 9 1734 2758 |
|  | Norway | Norwegian Institute of Public Health | Email: [kare.baevre@fhi.no](mailto:kare.baevre@fhi.no) |
|  | Austria | Statistics Austria | Email: [info@statistik.gv.at](mailto:info@statistik.gv.at) |
|  | France | French National Institute for Health and Medical Research & French National Institute for Statistics (INSEE) | <https://www.inserm.fr>  <https://www.insee.fr/en/information/3974508> |
|  | England and Wales | Office for National Statistics | www.ons.gov.uk |
|  | Czech Republic | Czech Statistical Office | DEATHS: Demographic Yearbooks are available in excel format on the Czech website  <https://www.czso.cz/csu/czso/casova_rada_demografie> |
|  | Estonia | Statistics Estonia | Email: stat@stat.ee |
|  | Hungary | Hungarian Central Statistical Office | https://kapcsolat.ksh.hu/ContactCenter/index.xhtml?lang=en |
|  | Lithuania | Lithuanian Department of Statistics | Email: aleksandra.golubovic@stat.gov.lt |
| Smoking and obesity | New Zealand | Statistics New Zealand | https://www.stats.govt.nz/integrated-data/apply-to-use-microdata-for-research/ |
|  | Finland | Statistics Finland | http://www.stat.fi/tup/mikroaineistot/index_en.html |
|  | Norway | Norwegian Institute of Public Health | Email: [kare.baevre@fhi.no](mailto:kare.baevre@fhi.no) |
|  | Austria | Statistics Austria | Email: [info@statistik.gv.at](mailto:info@statistik.gv.at) |
|  | France | French National Institute for Health and Medical Research & French National Institute for Statistics (INSEE) | <https://www.inserm.fr>  <https://www.insee.fr/en/information/3974508> |
|  | England and Wales | Office for National Statistics | www.ons.gov.uk |
|  | Czech Republic | Czech Statistical Office | Email: infoservis@czso.cz |
|  | Estonia | National Institute for Health Development, Estonia | Email: tai@tai.ee |
|  | Hungary | Hungarian Central Statistical Office | https://kapcsolat.ksh.hu/ContactCenter/index.xhtml?lang=en |
|  | Lithuania | Lithuanian Department of Statistics | Email: aleksandra.golubovic@stat.gov.lt |

# References

1 Mackenbach, J. P. *et al.* Trends in inequalities in premature mortality: a study of 3.2 million deaths in 13 European countries. *J Epidemiol Community Health* **69**, 207-217 (2014).

2 Hu, Y. *et al.* The Impact of Tobacco Control Policies on Smoking Among Socioeconomic Groups in Nine European Countries, 1990-2007. *Nicotine Tob Res* **19**, 1441-1449, doi:10.1093/ntr/ntw210 (2017).

3 Hoffmann, K. *et al.* Trends in educational inequalities in obesity in 15 European countries between 1990 and 2010. *Int J Behav Nutr Phys Act* **14**, 63, doi:10.1186/s12966-017-0517-8 (2017).
