# Supplementary figures and images for: Changing social inequalities in smoking, obesity and cause-specific mortality: Cross-national comparisons using compass typology
Source: PLoS One. 2020 Jul 10;15(7):e0232971. doi: 10.1371/journal.pone.0232971 (PMC7351173; doi:10.1371/journal.pone.0232971)

Fig S1: Trends in age-standardised smoking prevalence, all education groups combined, 30-79 year olds 1980-2010

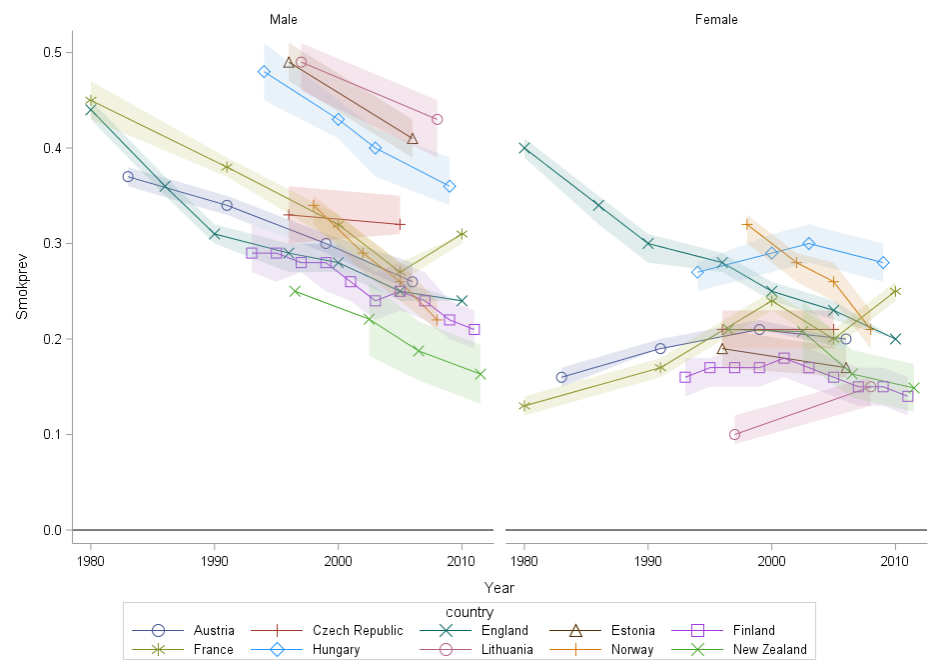

Supplement: S1 Fig — (PDF) [file pone.0232971.s010.pdf]

Fig S2: Trends in age-standardised obesity prevalence, 30-79 year olds 1980-2010

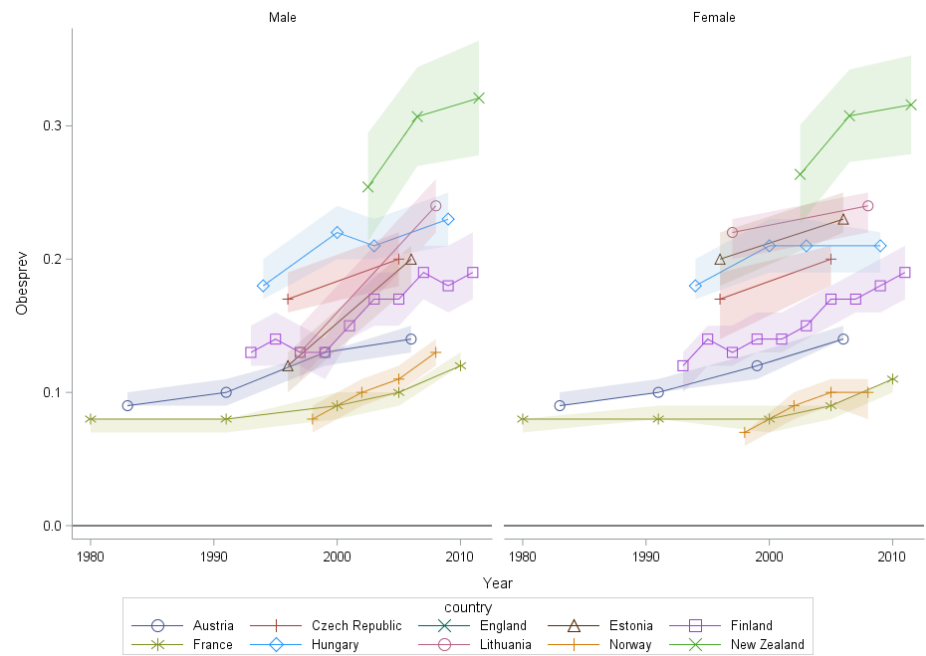

Supplement: S2 Fig — (PDF) [file pone.0232971.s011.pdf]
