# Supplementary material for: Changing social inequalities in smoking, obesity and cause-specific mortality: Cross-national comparisons using compass typology
Source: PLoS One. 2020 Jul 10;15(7):e0232971. doi: 10.1371/journal.pone.0232971 (PMC7351173; doi:10.1371/journal.pone.0232971)

Figure S3: Age-standardized mortality rates of CVD, cancer, external mortality, smoking-related mortality, in men (left) and women (right) 35-79 years

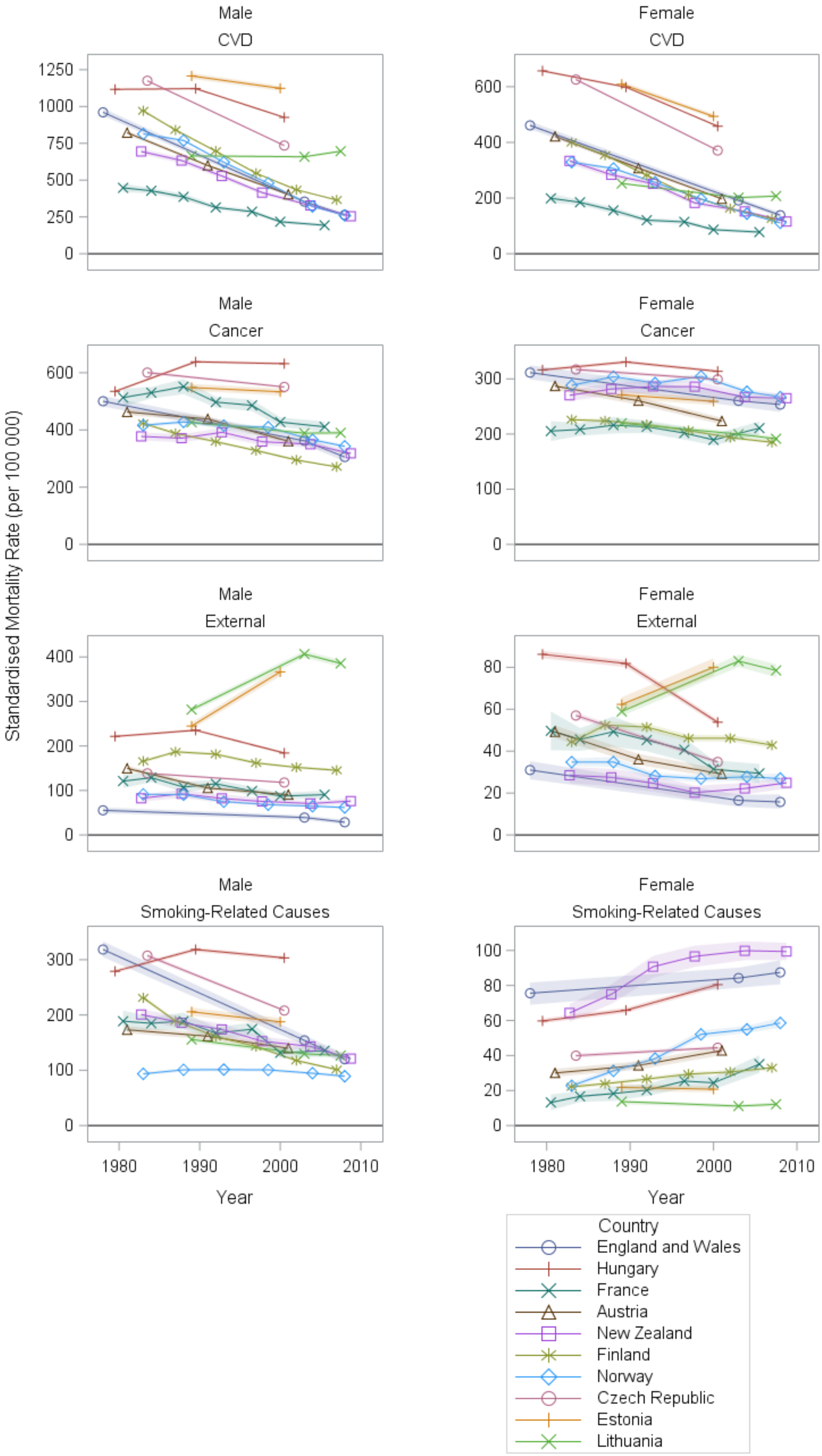

Supplement: S3 Fig — (PDF) [file pone.0232971.s012.pdf]
